# Supplementary material for: Structural insights into the nucleic acid remodeling mechanisms of the yeast THO-Sub2 complex
Source: eLife. 2020 Nov 16;9:e61467. doi: 10.7554/eLife.61467 (PMC7744097; doi:10.7554/eLife.61467)
Supplement: Supplementary file 1. [file elife-61467-supp1.docx]

**Table 1. Cryo-EM and model statistics**

|  | THO-Sub2  highRes  (EMDB-11859)  (PDB 7APX) | THO-Sub2  dimer  (EMDB-11871)  (PDB 7AQO) | THO-Sub2  bottom protomer  (EMDB-11871) | THO-Sub2  top protomer  (EMDB-11871) |  |
| --- | --- | --- | --- | --- | --- |
| **Data collection and processing** |  |  |  |  |  |
| Magnification | 64,000 x | | | |  |
| Voltage (kV) | 300 | | | |  |
| Electron exposure (e–/Å^2^) | 55.0 | | | |  |
| Defocus range (μm) | 0.5-3.0 | | | |  |
| Pixel size (Å) | 1.38 | | | |  |
| Symmetry imposed | C1 | | | |  |
| Initial particle images (no.) | 922,935 | | | |  |
| Final particle images (no.) | 298,657 | 113,076 | | |  |
| Map resolution (Å)  FSC threshold | 3.40  0.143 | 3.93  0.143 | 3.69  0.143 | 4.01  0.143 |  |
| Map sharpening *B* factor (Å^2^) | -108.6 | -81.8 | -71.7 | -73.6 | |
|  |  |  |  |  |  |
| **Refinement** |  |  |  |  |  |
| Initial model used (PDB code) | *de novo,* 5SUP | THO-Sub2  highres |  |  |  |
| Model resolution (Å)  FSC threshold | 3.5  0.143 | 4.5  0.143 |  |  |  |
| Model composition  Non-hydrogen atoms  Protein residues  Ligands | 20574  2889  0 | 41173  5783 |  |  |  |
| *B* factors (Å^2^)  Protein  Ligand | 135.92 | 600 |  |  |  |
| R.m.s. deviations  Bond lengths (Å)  Bond angles (°) | 0.006  0.739 | 0.010  1.026 |  |  |  |
| Validation  MolProbity score  Clashscore  Poor rotamers (%) | 2.6  8.0  2.5 | 3.1  25  3 |  |  |  |
| Ramachandran plot  Favored (%)  Allowed (%)  Disallowed (%) | 93.5  6.35  0.18 | 92.63 6.85  0.32 |  |  |  |
